# *PARK2* Mediates Interleukin 6 and Monocyte Chemoattractant Protein 1 Production by Human Macrophages

**DOI:** 10.1371/journal.pntd.0002015

**Published:** 2013-01-17

**Authors:** Louis de Léséleuc, Marianna Orlova, Aurelie Cobat, Manon Girard, Nguyen Thu Huong, Nguyen Ngoc Ba, Nguyen Van Thuc, Richard Truman, John S. Spencer, Linda Adams, Vu Hong Thai, Alexandre Alcais, Erwin Schurr

**Affiliations:** 1 McGill Centre for the Study of Host Resistance, The Research Institute of the McGill University Health Centre, Montreal, Quebec, Canada; 2 Departments of Human Genetics and Medicine, McGill University, Montreal, Quebec, Canada; 3 Hospital for Dermato-Venereology, Ho Chi Minh City, Vietnam; 4 National Hansen's Disease Program, LSU School of Veterinary Medicine, Baton Rouge, Louisiana, United States of America; 5 Department of Microbiology, Immunology and Pathology, College of Veterinary Medicine and Biomedical Sciences, Colorado State University, Fort Collins, Colorado, United States of America; 6 Laboratoire de Génétique des Maladies Infectieuses, Institut National de la Santé et de la Recherche Médicale, U980, Paris, France; 7 Université Paris René Descartes, Sorbonne Paris Cité, Faculté Médicine Necker, Paris, France; 8 St. Giles Laboratory of Human Genetics of Infectious Diseases, Rockefeller Branch, The Rockefeller University, New York, New York, United States of America; Institute of Tropical Medicine (NEKKEN), Japan

## Abstract

Leprosy is a persistent infectious disease caused by *Mycobacterium leprae* that still affects over 200,000 new patients annually. The host genetic background is an important risk factor for leprosy susceptibility and the *PARK2* gene is a replicated leprosy susceptibility candidate gene. The protein product of *PARK2*, Parkin, is an E3 ubiquitin ligase that is involved in the development of various forms of Parkinsonism. The human macrophage is both a natural host cell of *M. leprae* as well as a primary mediator of natural immune defenses, in part by secreting important pro-inflammatory cytokines and chemokines. Here, we report that down-regulation of Parkin in THP-1 macrophages, human monocyte-derived macrophages and human Schwann cells resulted in a consistent and specific decrease in interleukin-6 (IL-6) and monocyte chemoattractant protein 1 (MCP-1/*CCL2*) production in response to mycobacteria or LPS. Interestingly, production of IL-6 at 6 hours by THP-1 cells stimulated with live *M. leprae* and *M. bovis* BCG was dependent on pretreatment with 1,25-dihydroxyvitamin D_3_ (VD). Parkin knockdown in VD-treated cells blocked IL-6 induction by mycobacteria. However, IκB-α phosphorylation and levels of IκB-ξ, a nuclear protein required for IL-6 expression, were not affected by Parkin silencing. Phosphorylation of MAPK ERK1/2 and p38 was unaffected by Parkin silencing while JNK activation was promoted but did not explain the altered cytokine production. In a final set of experiments we found that genetic risk factors of leprosy located in the *PARK2* promoter region were significantly correlated with *M. leprae* sonicate triggered *CCL2* and *IL6* transcript levels in whole blood assays. These results associated genetically controlled changes in the production of MCP-1/*CCL2* and IL-6 with known leprosy susceptibility factors.

## Author Summary

Leprosy is an infectious disease with a strong host genetic component. The identification of host genetic lesions predisposing to disease is a powerful approach for mapping key junctions in the host pathogen interplay. Genetic variants located in the promoter region of the *PARK2* gene are replicated leprosy susceptibility factors. To better understand a possible contribution of *PARK2* to host effector mechanisms in leprosy patients, we developed a cellular model to test the contribution of the *PARK2* encoded parkin protein to host responses to mycobacterial antigens. We observed that parkin was a mediator of IL-6 production in response to mycobacterial antigen in both THP-1 macrophages and human Schwann cells while human monocyte-derived macrophages needed to be pre-activated with VitD to show the same impact. Parkin also impacted on the constitutive production of MCP-1. The regulatory activity of parkin on cytokine production was found to be independent of the canonical TLR-NFκB signalling pathway. We also tested association of *IL6* and *CCL2* gene expression levels in whole blood assays with *PARK2* polymorphisms. For both cytokines, we found significant associations with those *PARK2* variants that were established leprosy susceptibility factors. Hence, our results show that genetic *PARK2* variants that are correlated with leprosy susceptibility are also correlated with production of these cytokines following stimulation with *M. leprae* sonicate.

## Introduction

Leprosy, also called Hansen's disease, is a persistent infectious disease caused by *Mycobacterium leprae*. While leprosy can be transmitted by armadillos in the Southern United States [1] humans are the most important reservoir for *M. leprae* and the more common route is human-human transmission. Leprosy can lead to the destruction of peripheral nerves and subsequent extreme deformities of the skin and peripheral limbs. Despite effective chemotherapy and active case finding, the global number of new leprosy cases is estimated at over 200,000 annually [2]. Furthermore, microbiologically cured patients require follow-up to prevent nerve damage from sequellae of the disease. Due to the extreme social stigma and the long term follow-up, leprosy presents a large emotional and financial burden for affected communities and health care systems.

There is strong evidence for an important role of host genetics on leprosy susceptibility. For example, disease concordance is significantly higher among monozygotic as compared to dizygotic twins [3], and a number of complex segregation analyses have provided evidence for the presence of major genes in leprosy susceptibility [4]. A recent genome-wide association study detected and replicated susceptibility factors in *CCDC12*, *C13orf31*, *NOD2*, *TNFSF15*, *RIP2K*, and the *HLA-DR/DQ* locus and revealed a striking overlap with Crohn's disease susceptibility factors [5], [6]. Likewise, a number of candidate gene association studies have implicated additional genes in leprosy susceptibility [7]. Employing a genome-wide linkage based strategy, two major susceptibility loci were identified on chromosome 6 in Vietnamese families [8]. The locus on the short arm of the chromosome led to the identification of MHC class I and class III leprosy susceptibility factors [9], [10] while genetic variants in the regulatory region of *PARK2/PACRG* on chromosome 6q25 were uncovered as a common risk factor for leprosy [8]. The role of *PARK2* as susceptibility gene in infectious diseases was further supported by the subsequent identification of *PARK2* promoter variants as risk factors for typhoid fever [11].

The identification of *PARK2* as leprosy susceptibility gene was unexpected and the role of the *PARK2* encoded protein, Parkin, in leprosy pathogenesis has remained unknown. Parkin is an E3 ubiquitin ligase which is malfunctioning in autosomal recessive juvenile parkinsonism (AR-JP) [12]–[14]. Parkin has been given multiple roles related to neuronal survival in the context of Parkinsonism [15]–[22]. However, none of these functions explains the identification of *PARK2* as a genetic susceptibility factor for leprosy or typhoid fever, diseases characterized by the infection of macrophages with intracellular bacteria. Parkin/PACRG have been shown to be important for the autophagic elimination of aggregated proteins [21], [23], [24]. Interestingly, a role in autophagy is also found with immune regulators such as *NOD2* and other genes associated with both Crohn's disease and mycobacterial infections (reviewed in [25]). As first step towards the elucidation of Parkin in leprosy pathogenesis, we studied the impact of Parkin on measures of innate immunity in human macrophages and Schwann cells which are the main host cells of *M. leprae* in humans. We report here that abrogation of *PARK2* in macrophages and Schwann cells affects their ability to produce IL-6 and MCP-1, two key pro-inflammatory cytokines. Moreover, we demonstrate significant correlation of *IL6* and *CCL2* transcript levels in a whole blood assay with specific variants of *PARK2* previously identified as leprosy risk factors.

## Materials and Methods

### Human subjects

For the gene expression experiments, 62 unrelated Vietnamese Kinh individuals were recruited at the Dermato-Venereology (DV) Hospital in Ho Chi Minh City. Of those, 56 were leprosy patients while six had no history of leprosy disease. Since leprosy shows a strong gender bias, 43 subjects were males and 19 were females. Healthy Caucasian volunteers were enrolled for derivation of monocyte-derived macrophages. The study was conducted according to the principles expressed in the declaration of Helsinki. Signed informed consent was obtained from every person participating in the study. The study and subject enrolment were approved by the Ethical and Scientific Committee of the Dermato-Venerology Hospital and the Health Services Peoples' Committee, Ho Chi Minh City, Viet Nam, and the Research Ethics Board at the McGill University Health Centre, Montreal, QC, Canada.

### Reagents and antibodies

RPMI-1640, GlutaMAX, penicillin, streptomycin, fetal bovine serum (FBS), TRIzol, and AlexaFluor 488 anti-mouse were purchased from Invitrogen (Carlsbad, CA). Middlebrook 7H9 and ADC enrichment were purchased from BD Biosciences (Mississauga, ON, Canada). Phorbol 12-myristate 13-acetate (PMA), 4′,6-diamidino-2-phenylindole (DAPI), 1,25-dihydroxyvitamin D_3_ (VD), anti-Parkin monoclonal antibody (clone PRK8) and monoclonal anti-beta actin were purchased from Sigma-Aldrich (St-Louis, MO). Antibodies against IκBζ, phospho-IκBα (S32), phospho-ERK1/2, phospho-JNK, phospho-p38 and total JNK were purchased from New England Biolabs (Pickering, ON). PI3Kγ inhibitor AS605240 was from Santa Cruz Biotechnology (Santa Cruz, CA). HRP-conjugated goat anti-mouse and anti-rabbit antibodies were from Pierce Biotechnology (Rockford, IL). Recombinant human MCP-1 was purchased from R&D Systems (Minneapolis, MN).

### Cell lines and primary cells

THP-1 promonocytic cells were obtained from the American Type Culture Collection (Manassass, VA) and cultured in RPMI supplemented with 10% fetal bovine serum, GlutaMAX and penicillin-streptomycin antibiotic cocktail (Invitrogen). Primary human Schwann cells were purchased from Sciencell (San Diego, CA) and cultured in SCM supplemented with 5% serum and growth supplements (Sciencell). For stimulation and cytokine determination, Schwann cell medium was replaced with RPMI+10% FBS. THP-1 cells were differentiated into macrophages by incubating with 200 nM PMA for 24 h, then leaving the adherent cells to recover and differentiate for another 24 hr in medium without PMA. For some experiments, 10 nM VD was added to culture medium during and after PMA treatment. For human monocyte-derived macrophages, venous blood was collected from healthy volunteers. The PBMC fraction was isolated by Ficoll-Hypaque (Sigma) according to the manufacturer's instructions, diluted in RPMI medium and incubated for 1 hour onto gelatin-coated culture dishes. Adherent monocytes were trypsinized and transferred to a culture flask containing RPMI-10% FBS supplemented with 15% of L929 conditioned medium as a source of M-CSF. After 5 days, adherent, differentiated macrophages were collected and used for experiments. The purity was consistently >95% as determined by the expression of the macrophage mannose receptor. All cells were cultured in a humidified incubator at 37°C and 5% CO2. When *M. leprae* bacteria were added, cells were cultured under the same conditions except that antibiotics were omitted and the temperature was set to 33°C.

### Mycobacteria

Viable *Mycobacterium leprae* were obtained from the National Hansen's Disease Programs Laboratory at Louisiana State University (Baton Rouge, LA). The Thai-53 isolate of *M. leprae* was maintained in the footpads of athymic *nu/nu* mice and harvested as described previously [26]. Extraneous mouse tissue was removed by incubating the bacterial suspension in 0.1N NaOH for 3 min followed by extensive washing in RPMI 1640 (Gibco)+10% FCS (HyClone). Bacterial viability was determined by radiorespirometry, which measures the oxidation of ^14^C-palmitic acid to ^14^CO_2_ [27] and vital staining which measures cell wall integrity [28]. All *M. leprae* preparations underwent quality control testing for microbial contamination. Freshly harvested bacilli were stored at 4°C. Mice used in the propagation of *M. leprae* were housed in accordance with standards established in the PHS Guide to the Care and Use of Laboratory Animals (8th Edition) under a protocol approved by the IACUC of the National Hansen's Disease Program, Baton Rouge, La. (Assurance # A3032-1).

*M. leprae* whole cell sonicate was generated with support from the NIH/NIAID Leprosy Contract N01-AI-25469 at Colorado State University. Inactivated (irradiated) armadillo-derived *M. leprae* whole cells were probe sonicated with a Sanyo sonicator to >95% breakage to produce whole cell sonicate. *Mycobacterium bovis* BCG (Pasteur) and *M. tuberculosis* H37Ra were gifts of Dr. Marcel Behr (McGill University). They were cultured in Middlebrook 7H9 medium containing ADC supplement and used fresh to make single-cell suspensions.

### Gene knockdown by siRNA

STEALTH siRNA duplexes directed against Parkin and scrambled control duplexes were obtained from Invitrogen. siRNA sequences (sense, RNA) were as follows: Scrambled 5′-GGACUACAUGAUUCGACGUCAACUG-3′; Parkin_A 5′-GGAAACAUCAGUAGCUUUGCACCUG-3′; Parkin_B 5′-UUGCUUAGACUGUUUCCACUUAUAC-3′. Parkin A and B correspond respectively to positions 758–782 and 875–899 of human *PARK2* mRNA (NCBI accession BC022014.2). Cells were transfected with a Microporator device (NanoEnTek, Seoul, South Korea) following the manufacturer's instructions. Briefly, adherent cells were detached by trypsin digestion, rinsed, resuspended with a final concentration of 10 nM siRNA and electroporated with a single 20 ms pulse set at 1700 volts. Mortality due to electroporation was minimal, typically less than 10%. Cells were then returned to normal culture conditions for 48 hours, after which they were stimulated and assayed.

### Whole-blood assays

We collected 20 ml of whole blood from each individual by venipuncture. Blood samples were split in two aliquots and each aliquot was mixed 1∶2 with RPMI medium containing L-glutamine (300 mg/L) and HEPES (10 mM). One aliquot was stimulated with *M. leprae* sonicate at a concentration of 20 µg/ml, which approximately corresponds to an MOI of 50 *M. leprae* per white blood cell. The second aliquot was left untreated. Each aliquot, the stimulated one and the control, was divided into four 50 ml polystyrene tubes to facilitate better leukocytes adhesion and aeration of blood. Tubes were incubated for 30 hrs at 37°C, 5% CO_2_.

### RNA extraction

Total RNA from blood samples was extracted employing a modified protocol of the LeukoLOCK RNA extraction kit (Ambion, CA, USA). Briefly, blood aliquots were filtered by gravity through LeukoLOCK filters to isolate leukocytes. Collected cells were rinsed to eliminate red blood cells and lysed directly on the LeukoLOCK filters. Extraction of total RNA was performed according to the manufacturer's instructions. Isolated RNAs were kept under ethanol and ammonium acetate at −80°C. Prior to further experiments, all samples were cleaned with the RNeasy kit (Qiagen, Germany).

### Quantitative real-time PCR

The qualitative and quantitative analysis of RNA samples was done using Bioanalyzer 2100 (Agilent,USA). The QuantiTect Reverse Transcription kit (Qiagen, Germany) was utilized for reverse transcription of RNA samples. In brief, 500 ng of total RNA were treated with gDNA wipeout reagent to remove genomic DNA contamination and further transcribed following the manufacturer's instructions. Real-Time PCR was performed using the Rotor-Gene RG-3000 system (Corbett Research/Qiagen, Germany). The final volume of the PCR mix was 20 µl, with 16 ng of cDNA, 10 µl of Maxima Probe/ROX qPCR Master Mix (Fermentas, Lithuania), and 1 µl of *IL-6* or *MCP-1* TaqMan Gene Expression Assay probe mix (Applied Biosystems, USA). The *HPRT* gene probe was used as reference house-keeping gene. A comparative ΔΔCt method [29] was used to determine the regulation of the gene expression in response to *M. leprae* sonicate.

### Genotyping

Genomic DNA was obtained from all subjects enrolled in the study. We then obtained the genotypes for 31 SNPs that span approximately 320 kb of genomic DNA in the promoter region, exon 1 and intron 1 of *PARK2*. Based on the tag SNP information available from the International HapMap project database (www.hapmap.org/) for the Chinese population, which we know to strongly resemble the Vietnamese situation, these SNPs represent over 80% of the common genetic information of the target region. These SNPs were genotyped on the high-throughput SEQUENOM MassARRAY platform, which uses the iPLEX assay to incorporate mass-modified terminal nucleotides in the SBE step, which are then detected by MALDI-TOF MS [30] or the ultra-high throughput Illumina platform. This platform uses the GoldenGate assay followed by a bead-based technology to resolve individual SNP genotypes [31].

### Human cytokine ELISA

Macrophages were seeded into 24-well plates at a density of 5×10^5^ cells per well and Schwann cells at a density of 5×10^4^ cells per well. Cells were treated with stimuli for 6–24 hours; the supernatants were collected, cleared by centrifugation and assayed using Milliplex MAP 42-plex human cytokine kit on a Milliplex Analyzer 3.1 Luminex 200 machine (Millipore, Chicago, IL) according to the manufacturer's instructions. Alternatively, supernatants were analyzed using custom Q-Plex chemiluminescent multiplex ELISA arrays measuring human IL-1β, IL-6, IL-8, IL-10, MCP-1 and TNF (Quansys Biosciences, Logan, UT). Array images were acquired and analyzed using the Quansys Q-View imager and software.

### Western blot

THP-1 macrophages were cultured in serum-free medium overnight then treated with 100 ng/ml LPS for various times and lysed in denaturing buffer (8 M urea, 1% SDS, 40 mM Tris, pH 8.0). Twenty micrograms of protein were loaded onto a 12% polyacrylamide gel and transferred to a nitrocellulose membrane. The membrane was blocked with 1% casein in TBS and stained with antibodies overnight at 4°C then with HRP-conjugated goat anti-rabbit or anti-mouse for one hour. The signal was revealed with Immobilion Western substrate (Millipore, Billerica, MA). Equal loading was verified with an antibody against β-actin following reprobing of the membrane.

### Transcription factor ELISA

TransAM transcription factor assay kits for NF-κB and AP-1 families were obtained from Active Motif (Carlsbad, CA). The assays were used to measure the amount of nuclear protein that could bind to κB and AP-1 consensus oligonucleotides immobilized on a solid substrate. The bound nuclear proteins were probed with anti-p65 for NF-κB and anti-phospho-c-Jun for AP-1. In brief, macrophages were seeded into 6-well plates at 2×10^6^ cells per well and treated for 2 hours. Nuclear extracts were prepared according to the instructions given in the manual and 5 µg of nuclear proteins were used for the binding assays. Positive control extracts for NF-κB and AP-1 assays (5 µg of Raji or TPA-treated K562 extracts, respectively) were used to normalize the values.

### Statistical analysis

The expression levels of *IL6* and *CCL2* in *M. leprae* sonicate stimulated and non-stimulated whole blood assays for all 62 subjects were used as quantitative traits in an eQTL analysis. Association between expression levels of *CCL2* and *IL6* and SNP alleles in *PARK2*/*PACRG* was tested by means of the Likelihood Ratio Test as implemented in the GENMOD procedure of the SAS software version 9.2 (SAS Institute, Cary, NC, USA) and assuming a dominant genetic model since this is the model providing best evidence of association with leprosy [32]. Due to the known strong impact of gender on leprosy risk, we adjusted the analysis for sex of subjects.

## Results

### Parkin knockdown in THP-1

**Figure 1:**
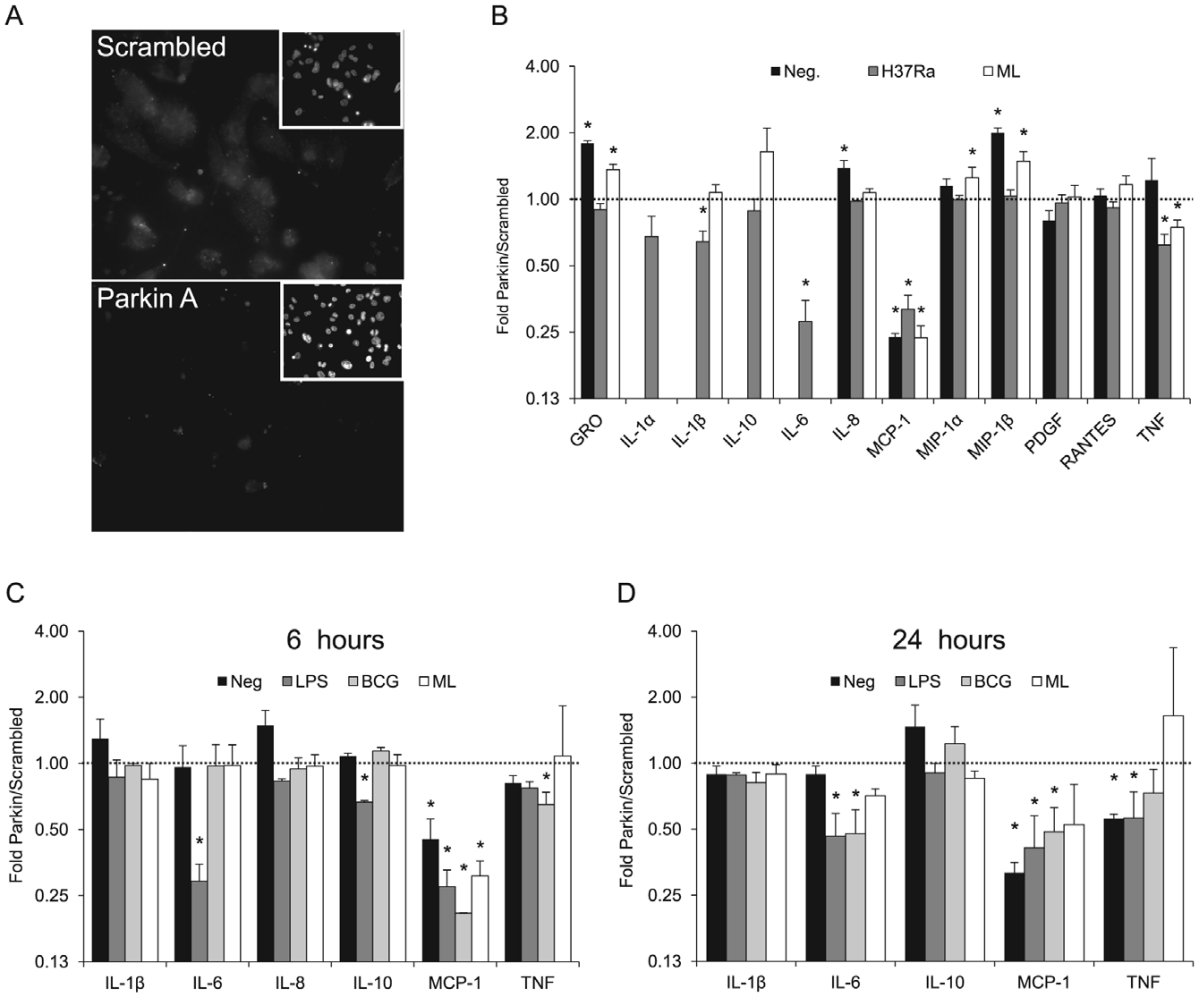
Parkin-silenced THP-1 macrophages cytokine screen. (**A**) Parkin was detected by indirect immunofluorescence of THP-1 cells following transfection with either scrambled siRNA (upper panel) or siRNA targeting Parkin (lower panel). Insets represent DAPI-stained nuclei. (**B**) PMA-differentiated THP-1 macrophages were transfected with control or Parkin-silencing siRNA. After 48 hours, cells were treated with H37Ra at an MOI of 10, *M. leprae* (ML) at an MOI 50, or left untreated (Neg.). After 6 hours, supernatants were collected and analyzed with a Milliplex 42-cytokine assay. Cytokines with detectable values (12 out of 42) are plotted on the graph. Cytokine production is expressed as ratio of cytokine secreted by cells transfected with siRNA for *PARK2* (Parkin) to cytokine secreted by cells transfected with control siRNA (scrambled). (**C**) PMA- differentiated THP-1 macrophages were transfected with control or Parkin-silencing siRNA. After 48 hours, cells were treated with LPS (10 ng/ml), *M. bovis* BCG at an MOI of 10, *M. leprae* (ML) at an MOI 50, or left untreated (Neg). After 6 hours supernatants were collected and analyzed with a Q-Plex custom cytokine multiplex assay. Values represent the ratio of concentrations produced by Parkin-silenced cells over controls ± SD of at least three independent experiments. (**D**) As described for **C** except that supernatants were collected after 24 hrs incubation with stimulants. * *p*<0.05, non-parametric t test of unpaired samples.

Parkin is expressed in many cells of the immune system, including macrophages and T-cells. We used siRNA-mediated *PARK2* gene knockdown to study the function of Parkin in macrophages. siRNA duplexes were delivered by high-efficiency electroporation into differentiated cells of the human acute monocytic leukemia cell line THP-1. Real-time quantitative PCR and indirect immunofluorescence analysis of Parkin showed that THP-1 cells transfected with Parkin-specific siRNAs expressed substantially less Parkin than THP-1 control cells transfected with a scrambled siRNA control (Figure 1A), with knockdown efficiencies ranging from ∼80% to 90% as determined by qPCR (data not shown).

### Parkin silencing downregulates secretion of IL-6 and MCP-1 in macrophages

To determine a possible role of Parkin in the production of soluble immune mediators by macrophages, THP-1 cells were differentiated into macrophages, transfected with siRNA (either Parkin A or scrambled control) and stimulated with live *M. tuberculosis* H37Ra or viable *M. leprae* (ML) for six hours (Figure 1B). A multiplex quantitation of 42 soluble immune mediators using the Milliplex system was performed on the culture supernatants to identify cytokines modulated by Parkin knockdown. While *M. tuberculosis* H37Ra induced a robust cytokine response, ML had a much lower impact. Out of the 42 soluble factors, a total of 12 cytokines/chemokines could be detected either at *ex vivo* production levels or induced by at least one stimulus (Figure 1B). When expressed as the ratio of cytokine concentrations secreted by Parkin knocked-down cells over controls, we observed that IL-6, induced by H37Ra, and MCP-1 either at constitutive production levels or induced are both diminished approximately four-fold by Parkin knockdown. By contrast, all other cytokines are modulated less than two-fold. We could not detect baseline IL-6 or induced by ML under these conditions.

In a second round of experiments, we sought confirmationof these initial observations by screening with a more limited number of cytokines and a different assay system, the Q-Plex multiplex ELISA. We also considered it important to test a 24 hour time point. One limitation was that H37Ra induced visible toxicity in macrophages at six hours which resulted in significant cell death at 24 hours (data not shown) precluding its use as a reliable stimulant for kinetics studies. We instead opted for *Mycobacterium bovis* – Bacille Calmette Guerin (BCG) which was nontoxic under the same experimental conditions. Since it was possible that BCG would not induce the same magnitude and range of response as H37Ra, we also included LPS, a well-known macrophage stimulant, as a positive control. Six cytokines/chemokines were measured: IL-1β, IL-6, IL-8 (CXCL8), IL-10, MCP-1 (CCL2) and TNF (Figure 1C, D). Similar to the first round, a pattern emerged with IL-6 and MCP-1 being specifically repressed by Parkin silencing. At 6 hours, IL-6 was only affected employing LPS as stimulant since BCG and *M. leprae* induced little IL-6 secretion (Figure 1C). At 24 hours, both LPS and BCG induced secretion of IL-6which was repressed by Parkin knockdown for both stimulants (Figure 1D). All IL-8 values at 24 hours, even in the un-induced state, were above the highest standard and are thus not represented in the figure. The overall Parkin effect was less pronounced at 24 hours, possibly reflecting a time-dependent plateau effect for cytokine production (Figure 1C, D).

### Parkin silencing downregulates secretion of IL-6 and MCP-1 in VD macrophages and Schwann cells

**Figure 2:**
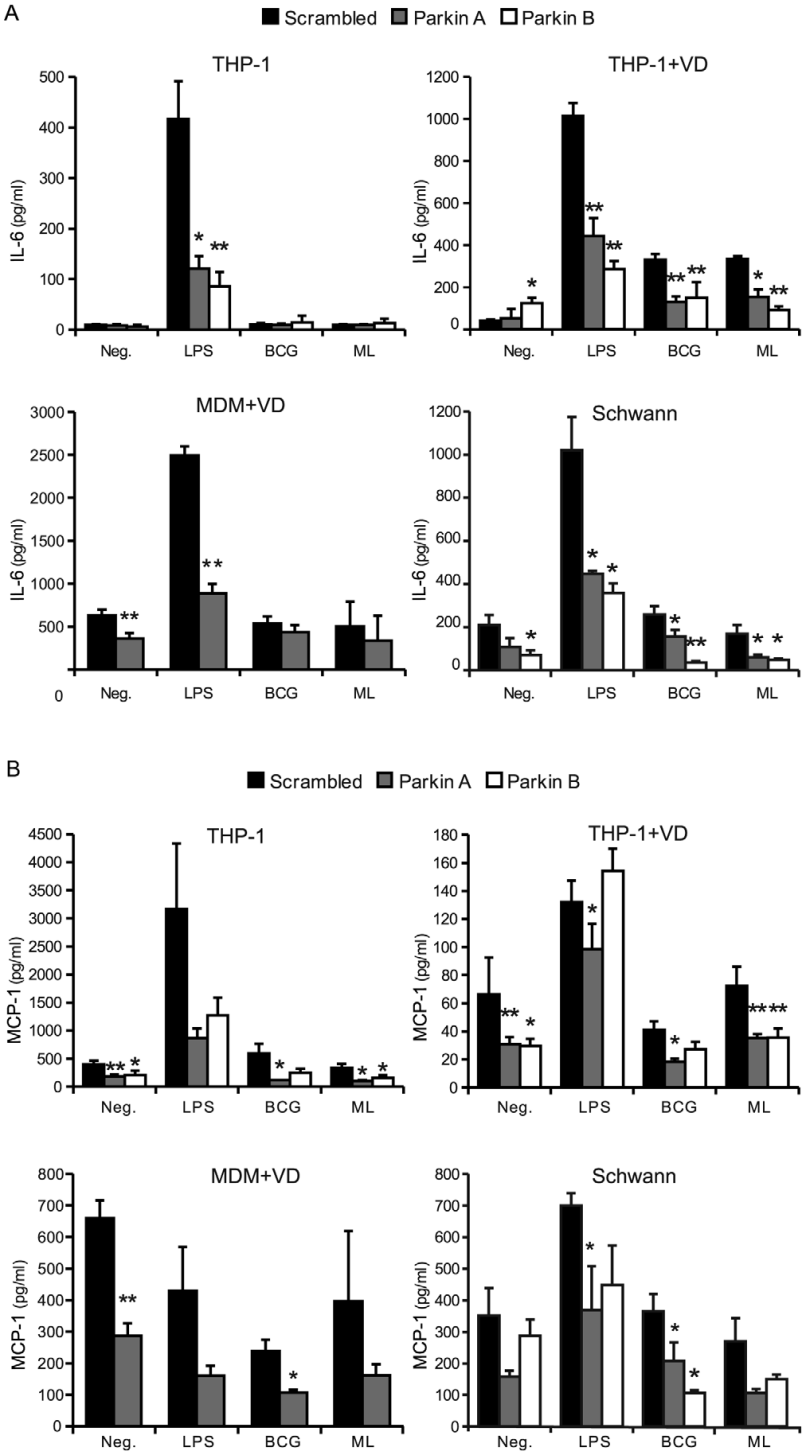
Parkin knockdown inhibits IL-6 and MCP-1 induction in macrophages and Schwann cells. PMA-differentiated THP-1 macrophages, VD-treated THP-1 macrophages, VD-treated human monocyte-derived macrophages and human Schwann cells were transfected with control or Parkin-silencing siRNA. Due to the limited number of cells available for each experiment, only one *PARK2* siRNA was used for MDM. After 48 hours, cells were treated with LPS (10 ng/ml), *M. bovis* BCG at an MOI of 10, *M. leprae* (ML) at an MOI 50, or left untreated (Neg.). Supernatants were collected after 6 hours and analyzed for (**A**) IL-6 and (**B**) MCP-1. The charts show the average concentration of cytokine in the supernatant in pg/ml ± SD of at least three independent experiments for each cell type. Scrambled: control siRNA, Parkin A: siRNA(A) for Parkin, Parkin B: siRNA(B) for Parkin. * *p*<0.05, ** *p*<0.01, non-parametric t test of unpaired samples.

In order to verify and extend the previous observations, a third round of experiments was carried out focusing on IL-6 and MCP-1 and adding a second distinct siRNA for Parkin knockdown, as well as cells of different types. Replication of the results with a second siRNA duplex was done to rule out off-target effects. While we had obtained experimental evidence for an effect of Parkin on IL-6 production in response to H37Ra and LPS, we could not detect a similar effect for BCG and *M. leprae* at 6 hours, presumably due to the low and delayed responsiveness of THP-1 macrophages to these mycobacteria. It had been proposed that priming of macrophages with the active form of vitamin D (1–25-hydroxyvitamin D_3_, VD) may increase the responsiveness of macrophages to microbial ligands [33] possibly due to the fact that VD can induce the expression of *NOD2*, an important sensor of intracellular bacteria [34]. We therefore primed THP-1 macrophages with 10 nM VD for the duration of the experiment and stimulated the cells with LPS, BCG or *M. leprae* and measured IL-6 and MCP-1 levels by ELISA (Figure 2A–B, top right panel). VD treatment strongly promoted IL-6 production by THP-1 macrophages in response to the mycobacteria (Figure 2A) and allowed the determination of the effect of Parkin knockdown on ML-induced IL-6. Parkin silencing with Parkin-A siRNA reduced ML-triggered IL-6 production to 48% of control (*p*<0.05) and to 35% (*p*<0.01) with Parkin B. However, VD treatment had no effect on the induction of MCP-1, which was still near baseline (Figure 2B, top right).

VD treatment did not prime human monocyte-derived macrophages to produce IL-6 in response to BCG or ML. These cells were not very sensitive to LPS either with only a fivefold increase in IL-6 (mostly repressed by Parkin knockdown) and no increase in MCP-1 production (Figure 2A–B, bottom left panel). Nonetheless, VD-treated MDMs spontaneously secreted substantial amounts of both cytokines which was significantly inhibited by Parkin knockdown (*p*<0.01).

Schwann cells are the primary host cells of *M. leprae* in human leprosy patients and express Parkin at levels similar to macrophages [8]. We transfected primary human Schwann cell cultures with siRNA and stimulated them for 6 hours as with macrophages. Both IL-6 and MCP-1 could be detected in the supernatants of Schwann cell cultures and were not affected by the presence of VD (data not shown). LPS induced a 5-fold induction of IL-6 and an approximate two-fold increase in the production of MCP-1 over baseline production levels. Neither BCG nor *M. leprae* stimulation resulted in a significant induction of either cytokines over resting levels. Parkin knockdown led to a general decrease of IL-6 and MCP-1 production, both at resting levels (inclusive the presence of BCG and ML) or induced by LPS (Figure 2A–B, bottom right panel).

### Parkin does not affect LPS-induced NF-κB signaling

**Figure 3:**
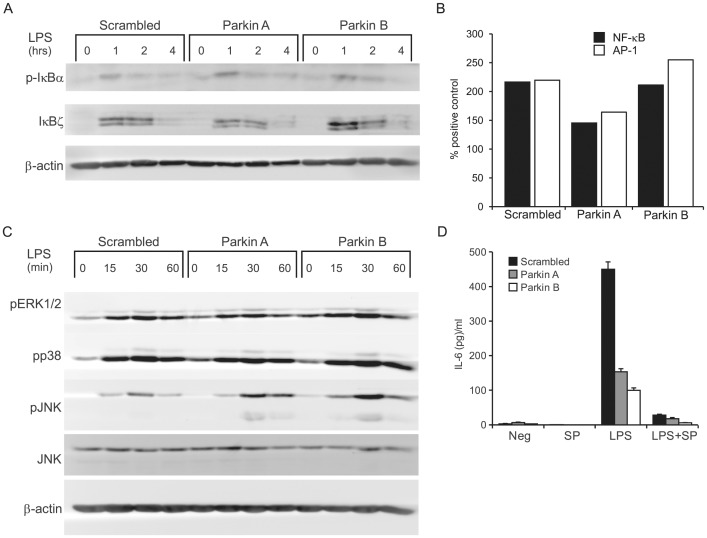
Parkin knockdown effect on NF-κB and MAPK signaling. (**A**) siRNA-transfected THP-1 macrophages were stimulated with 100 ng/ml LPS for the indicated times, then lysed and analysed by 12% SDS-PAGE followed by Western blotting. Membranes were stained for phospho-IκB-α then reprobed for IκB-ζ and β-actin (**B**) Nuclear extracts were also analyzed by transcription factor ELISA. THP-1 cells were treated with 100 ng/ml LPS for 4 hours, then extracted and analysed for NF-κB (p65) and AP-1 (phospho-c-Jun) binding to consensus DNA oligomers. Values are expressed as % binding of positive standard extracts and are representative of three experiments. Extracts from diluent (RPMI)-treated cells did not show DNA binding above background. (**C**) Membranes were stained for phospho-ERK1/2, phospho-p38 and phospho-JNK then reprobed for total JNK and β-actin. The JNK2 (p54) isoform is predominantly detected in these lysates although some phospho-JNK1 (p46) bands can be observed. Blots are representative of at least three experiments. (**D**) Transfected cells were pretreated with 30 µM JNK inhibitor SP600125 (SP) for 1 hour and then treated with LPS or diluent for 6 hours. IL-6 from the supernatants was measured by ELISA.

To resolve the apparent selectivity of Parkin in cytokine modulation, we measured IκB-α phosphorylation, a measure of canonical NF-κB activation, as well as the levels of the NF-κB-inducible IκB-ζ, a nuclear factor that associates with and activates a subset of NF-κB-dependent promoters upstream of genes such as *Il-6*, *Il-12b, Csf2* and *HBD2* [35], [36]. Western blotting of THP-1 lysates following Parkin knockdown and LPS stimulation did not reveal differences in phosphorylation of IκB-α or induction of IκB-ζ protein, strongly arguing against a role of these factors in the selectivity of Parkin immunomodulation (Figure 3A). Moreover, a transcription factor ELISA measuring NF-κB DNA-binding complexes in LPS-stimulated THP-1 nuclear extracts did not reveal a consistent effect of Parkin on transcriptional activity (Figure 3B).

### Effect of Parkin is independent of the mitogen-activated kinase pathway

Triggering of the MAPK cascade by TLR leads to the assembly of the AP-1 complex on cytokine/chemokine promoters and is essential for expression of most pro-inflammatory mediators. We measured the phosphorylation of the three major MAPK: ERK1/2, JNK and p38, in cells with normal or depleted Parkin levels and treated with LPS for 0–60 minutes (Figure 3C). ERK1/2 and p38 phosphorylation were not affected by Parkin. JNK phosphorylation was higher in knocked-down cells, which agrees with previous reports of Parkin suppressing JNK activity [37], [38]. To test a possible contribution of JNK to the abrogation of LPS-induced IL-6 production, we pretreated cells with a well-known JNK inhibitor, SP600125. Inactivation of JNK strongly suppressed IL-6 induction by LPS but failed to abolish the effect of Parkin silencing (Figure 3D). In addition, stronger JNK activation in the absence of Parkin did not translate into stronger AP-1 DNA binding, which was unaffected by *PARK2* knockdown (Figure 3B).

### PARK2 leprosy risk alleles are associated with MCP-1 and IL6 production

**Figure 4:**
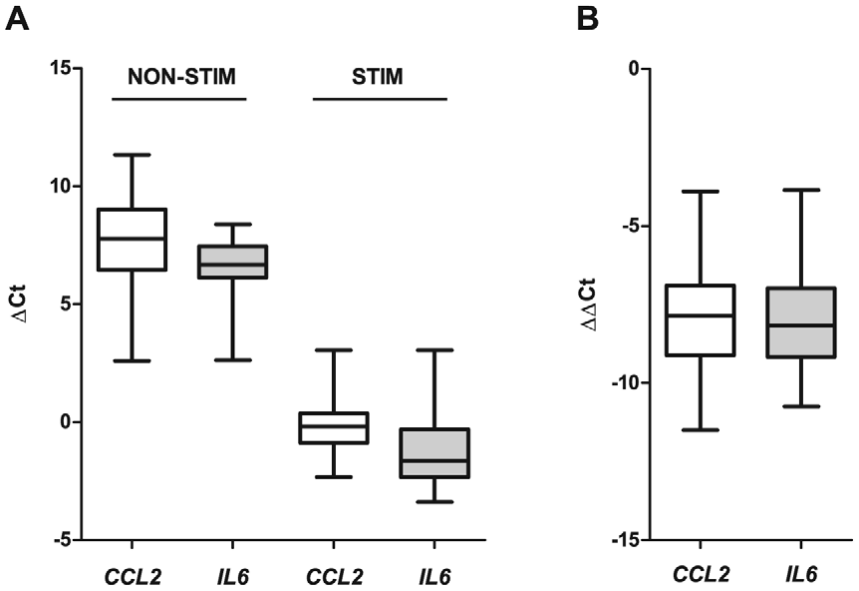
Whisker plots of *CCL2* and *IL6* transcript levels in whole blood cultures in the presence an absence of *M. leprae* sonicate. Whole blood from 62 Vietnamese subjects was stimulated with 10 µg/ml *M. leprae* sonicate and transcript levels of *CCL2* and *IL-6* were determined by real time PCR. (**A**) Transcript levels were normalized with the *HPRT* house keeping gene and expressed as ΔC_t_ in the absence (NON-STIM) and presence (STIM) of *M. leprae* sonicate. The median of the distribution is indicated by a solid line within the box. The resulting subdivision of the box indicates the distribution of the flanking 25% percentile in each direction while the error bars give the distribution of the upper and lower 25% of the ΔC_t_ values. (**B**) The increase of *CCL2* and *IL6* transcripts resulting from stimulation with *M. leprae* sonicate expressed as ΔΔC_t_. Plots as described in A.

While the above experiments provided strong evidence for a role of Parkin in MCP-1 and IL-6 production, they did not address the effect of different *PARK2* alleles on the production of these cytokines. Polymorphisms in the *PARK2* promoter region are strong risk factors for leprosy in the Vietnamese and the Brazilian populations [8]. To investigate a possible effect of genetic polymorphisms in the *PARK2* promoter region on *CCL2* and *IL6* transcript levels, we stimulated whole blood from 62 Vietnamese subjects with *M. leprae* sonicate and extracted total RNA for gene expression analysis. Whole *M. leprae* had low stimulation potential in the cellular assays. Hence, we opted for *M. leprae* sonicate as stimulant since it was reported to elicit a more robust immune response, presumably by exposing otherwise inaccessible antigens [39], [40]. We determined the gene expression levels before and after stimulation with *M. leprae* sonicate for both *CCL2* and *IL6* (Figure 4). In non-stimulated blood cultures both *CCL2* and *IL6* could be readily detected with *IL6* showing a slightly higher mean of transcript levels. Stimulation with *M. leprae* sonicate resulted in a strong upregulation of both genes. The extent of up-regulation and fold-induction of both genes were very similar (Figure 4).

*Ex vivo CCL2* and *IL6* transcript levels as well as the *M. leprae* sonicate triggered increase in *CCL2* and *IL6* transcripts were then correlated with a panel of SNPs that span the promoter region, exon 1 and part of intron 1 of the *PARK2* 5′ region. The selected SNPs capture more than 80% of the common genetic variation (allele frequency>5%) in the target region. Based on the existing linkage disequilibrium in the samples used for the analysis three groups of moderately correlated SNPs (r^2^>0.5 SNP bins) can be distinguished (Table 1). We detected significant evidence for association between SNPs of one bin and baseline levels of *CCL2* (ΔC_t_) as well as the increase of *CCL2* and *IL6* transcripts (ΔΔC_t_) following stimulation with sonicate (Table 1). Significant evidence for association was observed under a dominant effect of the major allele. The most consistent and strongest evidence for association was observed for a group of 3 SNPs (rs6915128, rs10806768, rs1333955) located approximately 60 kbp upstream of *PARK2* (Table 1). Interestingly, these were the same SNPs that had shown replicated evidence for association of their major allele with leprosy in both Vietnamese and Indian leprosy patients [32].

**Table 1:** Analysis of correlation between SNP genotypes and transcript expression in un-stimulated *ex vivo* whole blood cultures (ΔCt) or after *M. leprae* sonicate stimulation (ΔΔCt).

LD*	SNP	ΔCt CCL2		ΔCt IL6		ΔΔCt CCL2		ΔΔCt IL6	
		dominant major allele**	*P* *** (LRT)	dominant major allele	*P* (LRT)	dominant major allele	*P* (LRT)	dominant major allele	*P* (LRT)
sSNP	rs1954915	−1.344	0.217	−0.206	0.708	1.379	0.242	−0.367	0.703
sSNP	rs2023004	−0.407	0.434	−0.304	0.235	0.911	0.082	−0.070	0.868
BIN 1	rs9356034	0.337	0.538	−0.201	0.467	0.111	0.834	0.664	0.120
BIN 1	rs2846508	0.330	0.559	−0.212	0.456	0.111	0.838	0.597	0.178
sSNP	rs6936895	0.128	0.923	0.461	0.486	−0.027	0.981	−1.231	0.196
BIN 2	rs2803073	1.313	0.119	0.197	0.648	−0.671	0.422	0.216	0.756
BIN 3	rs2846463	−1.658	**0.049**	−0.186	0.665	1.511	**0.045**	0.859	0.166
BIN 2	rs6930532	1.369	0.206	0.328	0.552	0.046	0.968	−0.673	0.488
BIN 1	rs9365460	0.147	0.779	−0.012	0.963	−0.027	0.959	−0.118	0.791
BIN 3	rs1012423	−2.130	**0.002**	−0.507	0.145	1.540	**0.012**	0.567	0.268
BIN 1	rs2846556	0.143	0.781	0.043	0.870	−0.041	0.937	−0.230	0.600
BIN 2	rs2846524	1.378	0.206	0.306	0.575	0.046	0.968	−0.673	0.488
BIN 2	rs6455842	1.425	0.185	0.317	0.561	−0.053	0.964	−0.643	0.502
BIN 2	rs2846511	1.425	0.185	0.317	0.561	−0.053	0.964	−0.643	0.502
BIN 1	rs719650	−0.345	0.554	−0.252	0.391	0.820	0.144	0.316	0.493
BIN 1	rs2849536	−0.400	0.444	−0.282	0.283	0.709	0.158	0.498	0.225
BIN 1	rs10945859	0.337	0.531	0.052	0.848	−0.092	0.867	−0.155	0.738
BIN 1	rs10945860	0.337	0.531	0.052	0.848	−0.092	0.867	−0.155	0.738
BIN 2	rs2276201	1.335	0.115	0.193	0.653	−0.703	0.405	0.205	0.767
BIN 3	rs9356058	−1.149	0.139	0.003	0.994	1.511	**0.045**	0.859	0.166
BIN 2	rs7744433	1.559	0.096	0.240	0.613	−0.939	0.329	−0.084	0.916
BIN 2	rs7759501	0.736	0.695	0.875	0.353	0.386	0.818	−1.775	0.190
BIN 3	rs9458645	−1.149	0.139	0.003	0.994	1.511	**0.045**	0.859	0.166
BIN 3	rs6915128	−1.927	**0.003**	−0.396	0.242	1.820	**0.002**	1.107	**0.030**
BIN 3	rs10806768	−1.892	**0.009**	−0.290	0.440	1.881	**0.006**	1.121	**0.048**
BIN 3	rs1333955	−1.842	**0.006**	−0.299	0.389	1.828	**0.004**	1.075	**0.041**
BIN 2	rs1040079	1.335	0.115	0.193	0.653	−0.703	0.405	0.205	0.767
BIN 1	rs9456812	−0.004	0.995	−0.170	0.527	0.744	0.159	0.787	0.069
BIN 1	rs1333962	0.159	0.766	0.232	0.377	−0.540	0.323	−0.862	0.051

* LD: Linkage disequilibrium; singleton SNPs (sSNP) and correlated SNPs (BIN 1, BIN 2 and BIN 3) are indicated.

** Regression coefficients under dominant major allele model.

*** *P*-values for significance of correlation between genotypes and transcript levels under a dominant major allele model employing a likelihood ratio test (LRT); significant correlations are in bold and underlined.

We then asked if the major SNP allele was correlated with increased or decreased *CCL2* and *IL6* transcript levels. As can be seen from the regression coefficients in Table 1, in resting non-stimulated whole blood cultures the absence of the major alleles leads to a significant decrease in *CCL2* transcripts. The same trend is seen for *IL6* but fails to reach significance. Conversely, in *M. leprae* sonicate triggered cultures increase in both *CCL2* and *IL6* transcripts is significantly correlated with absence of the major allele. The most parsimonious explanation for this effect is that sonicate stimulation led to a plateau of both transcripts which was largely independent of the SNP genotypes. This resulted in a smaller increase in those cultures that already had a higher baseline transcript level. However, the most interesting aspect of these experiments was that the same alleles in the same SNPs impacted both *CCL2* and *IL6* transcript levels and susceptibility to leprosy.

## Discussion

Our results showed that Parkin participates in the modulation of IL-6 and MCP-1 production, two key mediators of innate immunity. *PARK2* knockdown in THP-1 macrophages, and Schwann cells consistently repressed LPS-induced IL-6 and basal MCP-1 levels. This repression displayed a degree of specificity as modulation of other cytokines or chemokines tested in our study was less pronounced. Nevertheless, we cannot rule out a significant effect of Parkin on other immune mediators that were either not included in our screen or not secreted by our cellular model. The preferential impact on certain immune mediators argued against a non-specific effect of Parkin on the general state of cellular physiology. Conversely, these results supported a Parkin-dependent modulation of specific pathways of immune responsiveness to microbial antigens. We hypothesized that the link between Parkin immune modulation and pathogen stimulation might be provided by Toll-like receptors (TLRs). Modulation of IL-6 and MCP-1 secretion via the TLR signaling cascade is a reasonable hypothesis since TLR recognition of *M. leprae* antigens is a well-established step in leprosy pathogenesis. In addition, genetic polymorphisms in TLR1 [41]–[43], *TLR2* [44], and *TLR4* [45] are associated with leprosy and/or leprosy reactions.

We evaluated the ability of Parkin-silenced cells to phosphorylate IκB-α, a critical step in the TLR pathway leading to activation of NF-κB, and found no difference of the IκB-α phosphorylation state. Parkin levels did not affect the induction of nuclear IκB-ζ, a positive regulator with some selectivity for the *IL6* promoter [35] that is partially under the control of NF-κB itself [36]. We also investigated the MAPK pathway which leads to the activation of the AP-1 transcriptional factor complex and found higher phosphorylation of c-Jun N-terminal kinase (JNK) after LPS treatment in Parkin-silenced cells, while ERK1/2 and p38 phosphorylation were unaffected. This agrees with previous reports of Parkin inhibiting JNK through mono-ubiquitination of Hsp70 [37], [38]. The role of JNK in modulating cytokine production by human macrophages is controversial. Some studies reported an inhibition of LPS-induced IL-12 expression by JNK in THP-1 cells [46], [47], while other studies showed the opposite [48]. However, we did not attempt to investigate this question in more detail since the impact of Parkin on IL-6 production was not related to its inhibition of JNK activation. Finally, a transcription factor ELISA of NF-κB and AP-1 complexes did not reveal any consistent effect of Parkin on LPS-induced DNA binding. While we failed to identify the mechanism by which Parkin modulates IL-6 and MCP-1 production, the results of our experiments argue against a general role of Parkin in the canonical TLR signaling cascade and are consistent with the more specific effect that Parkin exerts on IL-6 and MCP-1 production.

In our experiments, the strongest inducer of cytokines/chemokines was LPS and the impact of Parkin on host cell responsiveness was most easily detectable in response to this stimulus in all cell types tested. Consistent with previous reports *M. leprae* was a poor inducer of host responses [49], [50], [51]. Interestingly, the use of VD as a priming agent significantly boosted the ability of THP-1 cells to respond to mycobacteria with secretion of IL-6. Our results were similar to studies in human monocytes [49]. In these cells, BCG triggered production of IL-6 and MCP-1 while *M. leprae* triggered production of MCP-1 [49]. Why THP-1 cells would respond less to *M. leprae* is presently not known but it is possible that the dose of *M. leprae* used in our experiment (MOI = 50) was too low to promote MCP-1 production. Nevertheless, baseline production levels of MCP-1 and stimulus triggered production of IL-6 by Schwann and THP-1 cells were all diminished by Parkin knockdown. Given that both Schwann cells and THP-1 are sensitive to TLR2 ligands [52], [53] it appears that the immunogenic potential of BCG and *M. leprae* is dampened in intact bacteria.

Our functional studies identified PARK2 as mediator of IL-6 and MCP-1 production by macrophages. While this observation is exciting it did not address the impact of genetic *PARK2* polymorphisms on IL-6 and MCP-1 production. One efficient means to probe for a possible link between genetic variation and mechanistic phenotypes are so-called expression quantitative loci (eQTL) studies. We established a dense map of genetic markers of the 5′ region of the *PARK2* gene. These genetic markers were then correlated with *IL6* and *CCL2* transcript levels in whole blood assays in the presence and absence of high concentrations of *M. leprae* sonicate. We opted for high sonicate concentrations since in the functional studies whole *M. leprae* had relatively low potential to induce secretion of cytokines. A common set of three SNPs upstream of the *PARK2* promoter was significantly associated with induced *IL6* and *CCL2* and non-triggered *ex-vivo* transcript levels of *CCL2*. The same set of polymorphisms has recently been found to be key leprosy susceptibility factors in both Vietnamese and Indian leprosy patients [32]. The latter observation is important since pattern of linkage disequilibrium between Indians and Vietnamese differ substantially in the studied *PARK2* region suggesting a direct link between these SNPs and leprosy. Genome-wide eQTL studies have previously clearly shown that genetic variation at points far from the QTL, including different chromosomes (trans eQTL), can be significantly correlated with QTL expression levels [54]. While eQTL studies can tell us nothing about the mechanisms that cause the observed correlations, they do hold valuable information about which genes are likely to belong to the same host response pathway.

What is the possible functional relevance of Parkin modulating IL-6 and MCP-1 expression? IL-6 is a pluripotent cytokine and a key mediator of inflammation. IL-6 inhibits TGF-β dependent T_reg_ differentiation and induces differentiation of naïve T-cells to the IL-17-producing T_H_17 lymphocyte subset [55]. IL-17 is part of the anti-tuberculosis immune response via its role in organizing granulomas [56] and by inducing the T_H_17 generation of cathelicidin [57], a potent antimicrobial peptide [58]. Interestingly, a low T_H_17 response in tuberculosis patients correlates with decreased IL-6 receptor expression on CD4+ T cells [59]. Besides its role in the anti-mycobacterial immune response, IL-6 signaling is important for nerve regeneration and myelination following injury [60], cellular processes specifically disrupted by *M. leprae* [61], [62]. A conditional knockout of a major IL-6 family signal transducer (gp130) leads to Schwann cell degradation and peripheral nerve demyelination [63], which is consistent with reports of IL-6 promoting myelination in cultured Schwann cells [64], [65]. Just as IL-6, MCP-1 is a major immune regulator of granuloma formation in response to mycobacterial infections. In the mouse model of tuberculosis, *Ccl2* knock-out mice are significantly more susceptible to *M. tuberculosis* than their wild type littermates. In human genetic studies, evidence is accumulating for a role of MCP-1 in tuberculosis susceptibility possibly via the regulation of IL-12 levels [66], [67]. Less is known about MCP-1 in leprosy susceptibility. However, monocytes isolated from leprosy patients showed a reduced ability to produce MCP-1 in response to BCG and *M. leprae* as compared to healthy donors [51]. This observation is fully consistent with our results of (i) Parkin being a critical modulator of MCP-1 levels and (ii) the observed correlation of *CCL2* transcript levels with genetic markers in the *PARK2* promoter region that are confirmed leprosy susceptibility factors. Taken together, the present results provide an example of how genetic risk factors of leprosy may impact on leprosy pathogenesis by modulating the production of IL-6 and MCP-1, two key host response mediators.
